# Palliative care in humanitarian responses

**DOI:** 10.2471/BLT.25.295240

**Published:** 2026-06-01

**Authors:** Hanna Kaade, Felix Muehlensiepen, William E Rosa, Martin Heinze, Marcel Kamp, Matthew J Allsop

**Affiliations:** a Faculty of Health Sciences Brandenburg, Brandenburg Medical School Theodor Fontane, Universitätsklinikum Immanuel Klinik Rüdersdorf, Seebad 82/83, 15562 Rüdersdorf bei Berlin, Germany.; b User Experience in Digital Health Lab, Centre for Health Services Research, Brandenburg Medical School Theodor Fontane, Rüdersdorf bei Berlin, Germany; c Department of Psychiatry and Behavioral Sciences, Memorial Sloan Kettering Cancer Center, New York, United States of America.; d Leeds Institute of Health Sciences, University of Leeds, Leeds, England.

Humanitarian health responses are often judged by their ability to prevent avoidable death. However, even if deaths are avoided, crises also generate, amplify and prolong serious health-related suffering. First, by causing new life-threatening injury and acute illness. Second, by exacerbating physical and psychosocial symptom burdens among people with serious chronic and progressive conditions when treatment is interrupted and access to care is disrupted, fragmented or lost. Pain, breathlessness, anxiety, depression and functional loss are common after sustained injury in armed conflict, disasters and displacement. These effects are more pronounced where health and social care systems are already fragile, and where crisis further overwhelms, dismantles or destroys what services exist.^1^ Palliative care, the active, holistic care of people across all ages with serious health-related suffering due to severe illness, and especially of those near the end of life,^2^ should therefore be understood not as an optional add-on, but as an integral component of quality humanitarian health care. Consistent with World Health Assembly resolution WHA67.19,^3^ which recognizes palliative care as part of comprehensive care throughout the life course, humanitarian health responses should likewise be understood as incomplete if they do not include relief of serious health-related suffering.

The relevance of palliative care extends well beyond people who are dying. Palliative care is needed for people with traumatic injuries and complications; those living with advanced cancer and other serious illnesses such as organ failure or severe respiratory illness whose treatment is interrupted; people with serious infections or burns; older people with frailty, dementia and multimorbidity; and those in protracted crises, where fragmented systems and prolonged instability can intensify suffering over months or years. Palliative care also encompasses support for family caregivers, including psychosocial support for those carrying major caring responsibilities in conflict and displacement, where caregivers may themselves be traumatized and may in some cases be children. In these settings, unmet basic needs, cumulative trauma and poor continuity across fragmented services can further worsen suffering and outcomes, highlighting the importance of deliberate service design with palliative care integration in humanitarian health response planning.^4^

Existing policy and ethical frameworks already provide a basis for integrating palliative care into humanitarian health responses. The Sphere Handbook’s minimum standards recognize alleviation of suffering and palliative care as inherent to humanitarian health care, underscoring dignity and humane care as central to quality response.^5^ The World Health Organization (WHO) has also issued practical guidance on integrating palliative care and symptom relief into humanitarian emergencies and crises, including an essential package intended for responders and planners.^6^ In addition, WHO’s emergency medical teams initiative sets expectations for the content and quality of clinical services delivered by teams seeking classification and international recognition; palliative care and symptom relief should form part of the clinical standard within emergency medical teams' deployments.^7^

Despite these standards and guidance, palliative care is often missing in humanitarian response practice.^6^ This gap is frequently framed as a training deficit. Training matters, but implementation is also constrained by structural features of humanitarian health systems informed by a persistent false dichotomy between saving lives and relieving suffering, and assumptions that palliative care is a specialized, Western, resource-intensive addition. Donor priorities, accountability processes and financing can be misaligned with local needs and existing community caring practices.^8^ To address this situation, an implementation-focused approach that translates ethical commitment into operational practice is required. Building on established components of palliative care development,^9^ here we identify implementation barriers and propose a simple operational framework for humanitarian response.

First, essential medicines and supply chains are fragile, particularly in conflict contexts. Symptom relief depends on reliable access to a small selection of medicines (analgesics including opioids, antiemetics, anxiolytics, antipsychotics for delirium, antisecretory agents and antibiotics in selected cases) in addition to basic supplies such as dressings and oxygen where appropriate. Stockouts, import restrictions, security constraints and fragmented procurement can quickly render clinicians unable to provide relief, even when they have the skills to do so.

Second, service delivery models in crises often prioritize throughput and acute stabilization, with limited time and privacy for communication, goal-setting and family support. In mass-casualty settings, triage protocols appropriately focus on maximizing survival, but they also create a predictable group of patients for whom curative treatment is either not possible or not proportionate to their needs. Palliative care guidance for humanitarian emergencies addresses this reality and proposes integrating symptom relief and palliative care into response planning rather than leaving it to individual clinicians improvising at the bedside. Palliative care should therefore be understood as complementing, rather than replacing, appropriate life-saving and restorative care.

Third, health workers’ constraints and skill-mix problems are decisive. Humanitarian responses rely on teams rotating in and out, task-shifting and clinicians working outside their usual scope of practice. Even where specialist palliative care is unavailable, a generalist approach (basic symptom assessment, access to first-line palliative care medicines, skilled communication and family and caregiver support) can be delivered by a wide range of clinicians when protocols, supervision and referral pathways are in place. The question is not whether every setting can deploy specialists, but whether every response ensures that palliative care functions are covered and that staff have practical tools to safely provide adequate palliation (that is, symptom relief, comfort care and end-of-life support). 

Fourth, insufficient leadership and accountability for palliative care within humanitarian responses can leave clinicians without protocols, essential medicines (that is, opioids for moderate to severe pain) or organizational support for difficult conversations and decision-making. Under these constraints, moral distress is common when clinicians witness preventable suffering yet are unable to act in line with professional and ethical obligations.^10^ Strengthening palliative care as a core response function, supported by the availability of medicines, practical guidance and team supervision, can reduce moral distress by making symptom relief and communication routine and shared, rather than improvised and isolated. Such an approach supports staff well-being and helps sustain quality care over prolonged crises. Training should not only strengthen palliative care capability among humanitarian responders but also prepare the wider palliative care workforce to engage in humanitarian situations through appropriate practice, preparedness and familiarity with response systems, procedures and ethical frameworks.

Frontline experience suggests that these barriers to palliative care provision are both practical and conceptual. For example, a cross-sectional online survey of 114 Red Cross and Red Crescent Emergency Response Unit health delegates^11^ found strong endorsement of integrating palliative care into emergency response (71.1%; 81/114 delegates rated this extremely important). However, many lacked training: 82.7% (94) reported no prior palliative care training for humanitarian settings, and 91.4% (96/105) supported inclusion of specialized training in the curricula of the emergency response units.^11^ Reported barriers included lack of time or resources, absence of palliative care policies, cultural barriers and limited access to essential medicines such as morphine. Breaking bad news was common, with one in four respondents having done so during deployment, although most (72.1%; 62/86) had received no prior communication training, and a majority reported substantial challenges related to language barriers and cultural or religious differences.

A practical way to translate standards into implementation is to specify the minimum functions that humanitarian health actors should ensure, while recognizing that how these are delivered will vary across acute conflict, sudden-onset disasters and protracted crises. Box 1 presents a simple framework to guide implementation of palliative care in humanitarian responses^9^ and the logic of minimum standards for palliative care provision in humanitarian settings. The framework identifies core operational components, alongside family and community support and measurement functions that help sustain implementation over time. These components should be underpinned by community empowerment and context-relevant research and learning so that palliative care can be adapted, evaluated and strengthened across diverse crisis contexts.^9^

Box 1 **Operational components for implementing palliative care in humanitarian health responses^a^**

**Policy, leadership and accountability**

- Treat palliative care as a required element of quality humanitarian health care, not a discretionary add-on.
- Assign a palliative care focal point within the response and clarify roles and responsibilities.
- Embed symptom relief and palliative care within triage processes, clinical protocols and referral pathways (including clear escalation routes).

**Education and workforce capability**

- Build generalist palliative care capability across the humanitarian workforce.
- Train and support staff in rapid symptom assessment, first-line management and communication with patients and families.
- Provide supervision and decision support (on-site or remote where feasible) to enable safe good-enough palliation under pressure.
- Prepare the wider palliative care workforce to contribute in humanitarian situations, including through familiarity with relevant response systems, procedures, triage processes and operational constraints.

**Access to essential medicines and supplies**

- Ensure reliable access to a core set of symptom-relief medicines (including opioids for pain) and basic supplies required for comfort care and symptom management.
- Plan medicines access as a preparedness and governance issue: procurement routes, regulatory compliance, safe storage and dispensing, and last-mile logistics. This planning should include contingency arrangements for controlled medicines in emergencies, including simplified procedures and regulatory flexibility where permitted, alternative procurement routes and appropriate therapeutic alternatives where needed.
- Recognize that without medicines availability, training alone cannot translate into relief at the bedside.

**Service delivery integration**

- Integrate symptom relief and palliative care into emergency, trauma, inpatient and community pathways to avoid reliance on improvisation.
- Support continuity across transfers and handovers where possible, including through clear documentation.
- In mass casualty settings, ensure structured approaches for people for whom curative treatment is not possible or not proportionate, prioritizing comfort, dignity and family support, with clear decision-making frameworks, documentation and oversight to reduce the risk of inappropriate classification.

**Family, caregiver and bereavement support**

- Provide basic psychosocial support and caregiver guidance as routine elements of care in crises.
- Support culturally appropriate family, peer and community responses to caregiving, dying and bereavement, including refugee- and community-led networks where formal services are limited.

**Measurement, learning and quality improvement**

- Track relief of suffering with simple, feasible indicators, such as symptom assessment recorded; essential medicines available; communication with family or caregivers documented.
- Use simple learning and quality improvement cycles to adapt and strengthen delivery within and across responses, signalling that relief of suffering is part of core performance.

^a^ Organized according to core components of palliative care development, as outlined in WHO guidance on assessing palliative care delivery.^9^

This framework highlights that providing palliative care is not synonymous with delivering specialist services. Palliative care is about ensuring that relief from suffering is treated as a core clinical task and is operationalized in the same way that humanitarian actors organize infection prevention, triage, referral and supply chains. In many humanitarian settings, implementation will depend not only on formal clinical pathways but also on the capacities of families, communities and local networks of care. The framework also contributes to avoiding the conception that palliative care is only relevant when death is imminent. In practice, symptom relief and communication are essential across the continuum of severity and across phases of response, from the first days of an acute crisis to protracted displacement.

Strengthening these system components can also protect staff well-being. When preventable suffering cannot be relieved, moral distress may intensify; embedding palliative care through protocols, medicines, training and pathways can help by providing structured approaches to symptom relief and communication, and by normalizing team support around deaths that occur despite best efforts.^10^

Palliative care is embedded in the ethical foundations of humanitarian action: impartiality, dignity and the relief of suffering. The challenge is to ensure that these commitments are consistently translated into the everyday mechanics of emergency health response (medicines, protocols, skills, pathways and accountability). WHO guidance and humanitarian standards provide a workable basis for action, but frontline experience suggests that preparedness and delivery remain uneven. Embedding palliative care as a clinical standard across preparedness, response and recovery would improve the quality of health care in humanitarian settings, support families and communities, and reduce moral distress among staff. In crises, the ability to relieve suffering depends on systems, not improvisation. Palliative care is an essential, fundamental component of humanitarian health responses and a necessary part of closing the gap between need and delivery.
